# Investigation of periodontal disease development and *Porphyromonas gulae* FimA genotype distribution in small dogs

**DOI:** 10.1038/s41598-024-55842-8

**Published:** 2024-03-04

**Authors:** Junya Yasuda, Hidemi Yasuda, Ryota Nomura, Saaya Matayoshi, Hiroaki Inaba, Enrique Gongora, Naoki Iwashita, So Shirahata, Noriyuki Kaji, Tatsuya Akitomo, Chieko Mitsuhata, Jumpei Uchiyama, Tomoki Fukuyama, Michiyo Matsumoto-Nakano, Kazuhiko Nakano, Masaru Murakami

**Affiliations:** 1 Department of Molecular Biology, School of Veterinary Medicine, Azabu University, Sagamihara, Kanagawa Japan; 2 Yasuda Veterinary Clinic, Meguro, Tokyo, Japan; 3 Department of Pediatric Dentistry, Osaka University Graduate School of Dentistry, Suita, Osaka Japan; 4 Department of Pediatric Dentistry, Graduate School of Biomedical and Health Sciences, Hiroshima University, 1-2-3 Kasumi, Minami-ku, Hiroshima, 734-8553 Japan; 5 Department of Pediatric Dentistry, Okayama University Graduate School of Medicine, Dentistry and Pharmaceutical Sciences, Okayama, Japan; 6 Department of Pharmacology, School of Veterinary Medicine, Azabu University, Sagamihara, Kanagawa Japan; 7 Bioalch, Fuchu, Tokyo Japan; 8 Primo Animal Hospital, Sagamihara, Kanagawa Japan; 9 Department of Bacteriology, Okayama University Graduate School of Medicine, Dentistry and Pharmaceutical Sciences, Okayama, Japan

**Keywords:** Bacterial pathogenesis, Bacteriology

## Abstract

In dogs, *Porphyromonas gulae* is a major periodontal pathogen with 41-kDa proteins polymerizing to form a filamentous structure called fimbriae or pili, termed FimA. FimA is classified into three genotypes: A, B, and C, and there are combinations of types A, B, C, A/B, A/C, B/C, and A/B/C. Periodontal disease is the most common oral disease in small dogs, but the periodontal disease status and *P. gulae* colonization at each dog age and breed remain unclear. In this study, we stratified 665 small dogs and analyzed the periodontal status and distribution of *P. gulae* with each FimA genotype. Dogs with periodontal disease and FimA genotype tended to increase with age. The dogs with at least one FimA genotype had significantly more severe periodontal disease compared with *P. gulae*-negative dogs (*P* < 0.01). Additionally, periodontal status was significantly associated with specific FimA genotype distribution in Toy Poodles and Chihuahuas (*P* < 0.05), whereas there was no such association in Dachshunds. These results suggest that the onset of periodontal disease and *P. gulae* colonization are related and progress with age. The relationship between periodontal disease and FimA genotype may differ depending on the dog breeds.

## Introduction

Periodontal disease is a common inflammatory disease caused by bacterial infection^1,2^ and is found in more than 80% of adult dogs^3^. Periodontal disease begins with gingivitis, in which periodontopathic bacteria form a biofilm in the gingival sulcus between the teeth and gingiva, causing the gingival margin to become red and swollen^4^. As gingivitis becomes chronic, the inflammation gradually spreads to periodontal tissues such as the periodontal ligament and alveolar bone, forming irreversible deep periodontal pockets in the gingival sulcus^4^. As a result, healthy periodontal tissue is lost, resulting in mobility and loss of teeth^3^. Chronic periodontitis is also associated with systemic disease^5^.

The presence of highly periodontopathic bacterial species in biofilms formed in the gingival sulcus increases the risk of developing periodontitis due to dysbiosis caused by a microbial shift in the formed biofilm^6^. *Porphyromonas gulae* is a Gram-negative, black pigmented anaerobe and the major periodontal pathogen in dogs^7^. One of the most common virulence factors of *P. gulae* is a filamentous structure (FimA) polymerized by 41-kDa protein^7,8^. FimA is classified into three genotypes (A, B, and C) based on differences in putative amino acid sequences^8^. Of these FimA genotypes, *P. gulae* with type C fimbriae are the most virulent in periodontal disease^8^ and predominate in the oral cavity of dogs with severe periodontitis^8^.

Periodontal disease progression and tooth loss are associated with aging^9^. Recent studies have shown that the distribution of the FimA genotypes affects the number of remaining teeth in dogs over 8 years of age^10^. However, previous studies focusing on the FimA genotypes have used 100–200 dogs, and the degree of progression of periodontal disease (the leading cause of tooth loss) in dogs of various ages is unknown. In addition, dogs have seven FimA genotype distributions: types A, B, C, A/B, A/C, B/C, and A/B/C. Previous studies did not have enough samples to analyze these groups in detail, and many studies focused only on detecting three types: A, B, and C. Furthermore, no studies have compared and analyzed the periodontal condition and FimA genotypes, focusing on specific small dog breeds.

In the present study, we clarified the periodontal status and the FimA genotype distribution of *P. gulae* in 665 small breed dogs stratified by age group. We analyzed the relationships between periodontal status and the distribution of each FimA genotype. We also compared periodontal conditions and FimA genotype distribution among major small dog breeds, including Toy Poodle, Dachshund, and Chihuahua.

## Results

### Age distribution of periodontal disease status in small breed dogs

**Figure 1:**
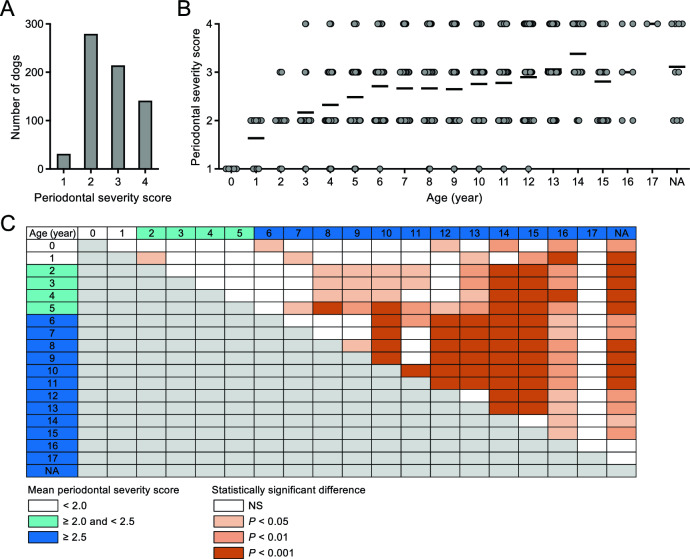
Severity of periodontal disease in small breed dogs. (**A**) Number of dogs with each periodontal severity score. (**B**) Age distribution of periodontal severity scores. Each circle represents the data of one dog, and horizontal bars represent the mean score at each age. (**C**) Statistical analysis of periodontal severity scores in each age group. White color for age indicates a mean periodontal severity score of < 2.0, light blue indicates ≥ 2.0 and < 2.5, and dark blue indicates ≥ 2.5. Light red color for each square indicates *P* < 0.05, medium red indicates *P* < 0.01, and dark red indicates *P* < 0.001.

When the periodontal disease status of 665 dogs {(1) no significant findings; (2) mild periodontal disease; (3) moderate periodontal disease; and (4) severe periodontal disease} was scored, the highest number of dogs had a score of 2 (n = 279, 42.0%), followed by a score of 3 (n = 214, 32.2%), 4 (n = 141, 21.2%), and 1 (n = 31, 4.6%) (Fig. 1A). The periodontal severity scores increased with age (Fig. 1B). All dogs under age 1 year had a score of 1, scores of 2 began appearing at 1 year, and scores of 3 and 4 began appearing at ages 2 and 3 years, respectively. Above age 3 years, dogs with a score of 4 were found in all age groups, whereas no dogs above age 13 years had a score of 1. The periodontal severity score increased markedly from age 0 to 6 years: all mean scores in the age groups from 0 to 5 years were < 2.5, whereas all mean scores in the age groups from 6 years and over were > 2.5. Statistical analysis showed that periodontal severity scores tended to increase significantly with age (Fig. 1C).

### Age distribution of the patterns of each FimA genotype in small breed dogs

**Figure 2:**
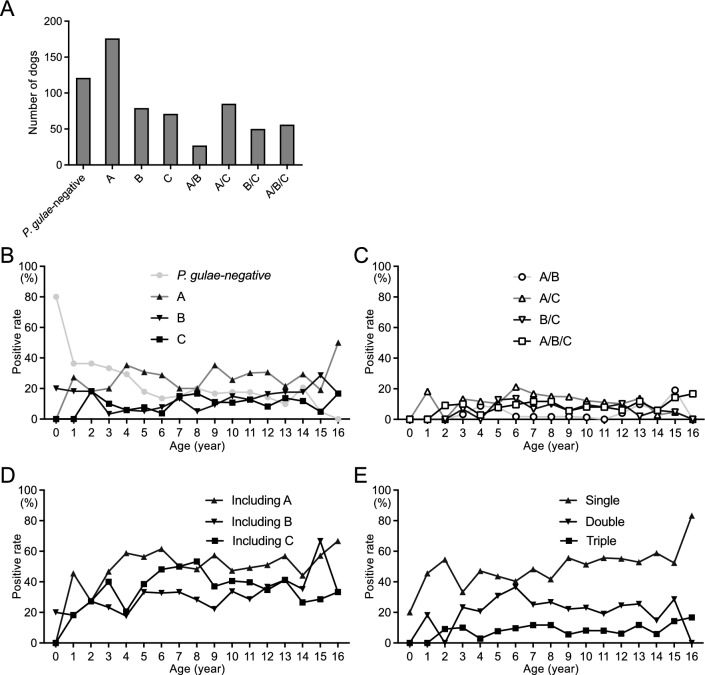
FimA genotype distribution in small breed dogs. (**A**) Number of dogs with each FimA genotype. Age distribution of FimA genotype: Single genotype (**B**), multiple genotypes (**C**), including FimA types A, B, and C regardless of single or multiple genotypes (**D**), and number of FimA genotypes (**E**).

Of 665 dogs, 544 (81.8%) were positive for *P. gulae*. In *P. gulae*-positive dogs, the FimA genotype distribution was classified into seven patterns: A (n = 176, 26.5%), B (n = 79, 11.9%), C (n = 71, 10.7%), A/B (n = 27, 4.1%), A/C (n = 85, 12.8%), B/C (n = 50, 7.5%), and A/B/C (n = 56, 8.4%) (Fig. 2A). The rate of *P. gulae*-negative dogs decreased with age, with a significant trend toward age (*P* < 0.001) (Fig. 2B). In dogs with a single genotype, type A was detected at age 1, type B at age 0, and type C at age 2. The detection rate of a single genotype at each age ranges from 0 to 50.0%, and all single genotypes had a significant trend with age (*P* < 0.001). Dogs with multiple genotypes began to be detected between 1 and 2 years of age. The detection frequency of each type at each age was less than 20% (Fig. 2C). The rate of dogs with type A, B, or C, regardless of whether the dogs had single or multiple genotypes. The rate of dogs with single, double, or triple FimA genotypes shows a significantly increasing trend with age (*P* < 0.001) (Fig. 2D,E).

### Relationship between FimA genotype distribution and periodontal severity score

**Figure 3:**
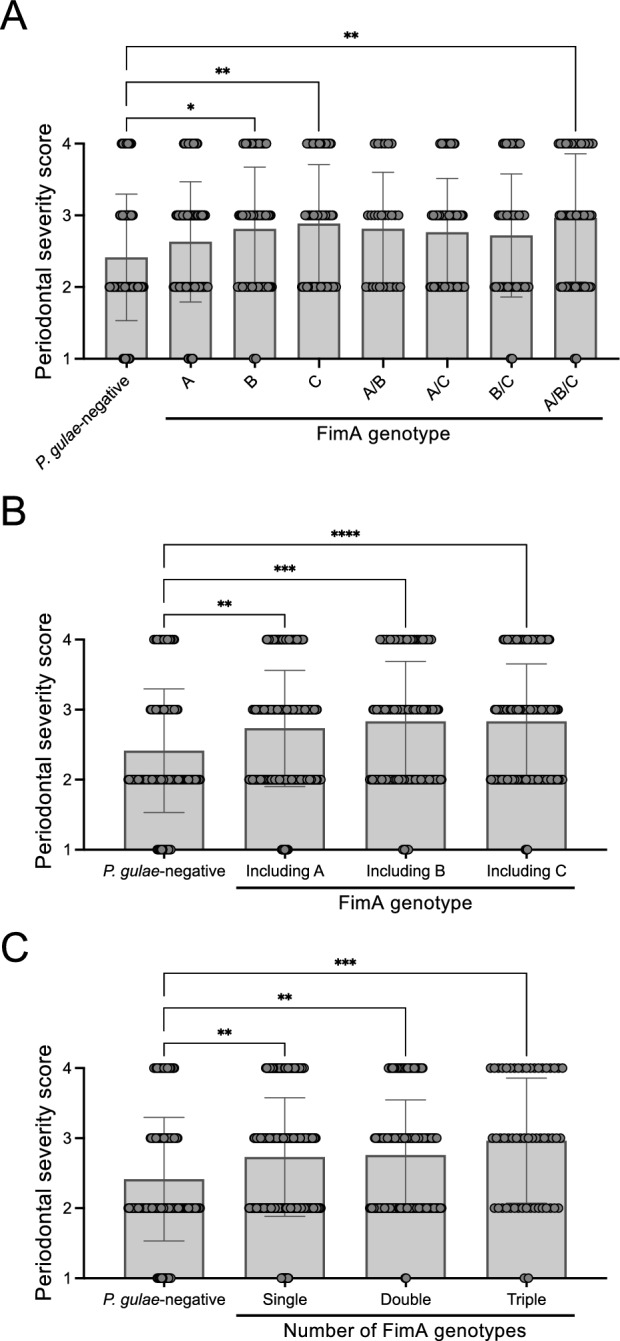
Association between severity of periodontal disease and FimA genotype distribution in small breed dogs. Severity of periodontal disease at each FimA genotype (**A**), including FimA types A, B, and C, regardless of single or multiple genotypes (**B**) and number of FimA genotypes (**C**). Each circle represents the data of one dog. Gray bars represent the mean periodontal severity score. Data expressed as the mean ± standard deviation. *P < 0.05, **P < 0.01, ***P < 0.001 between groups.

The periodontal severity score of the *P. gulae*-negative group (mean ± standard deviation; 2.4 ± 0.9) was lower than that of the groups with any FimA genotype distribution pattern (mean ± standard deviation; 2.7 ± 0.9 to 3.0 ± 0.9) (Fig. 3A). The periodontal severity score of the *P. gulae*-negative group was significantly lower than that of the FimA genotype B, C, and A/B/C groups. (*P* < 0.05). Next, *P. gulae*-positive groups were classified by having either A, B, or C FimA genotypes, regardless of whether they had single or multiple FimA genotypes. The periodontal severity score of the *P. gulae*-negative group was significantly lower than that of the groups containing any FimA genotype (mean ± standard deviation; 2.7 ± 0.8 to 2.8 ± 0.9) (*P* < 0.01) (Fig. 3B). When the *P. gulae*-positive group was separated into having single, double, and triple FimA genotypes, the periodontal severity score of the *P. gulae*-negative group was significantly lower than that of the group carrying any number of FimA genotypes (mean ± standard deviation; 2.7 ± 0.8 to 3.0 ± 0.9) (*P* < 0.01) (Fig. 3C).

### Comparison between Toy Poodles, Dachshunds, and Chihuahuas

**Figure 4:**
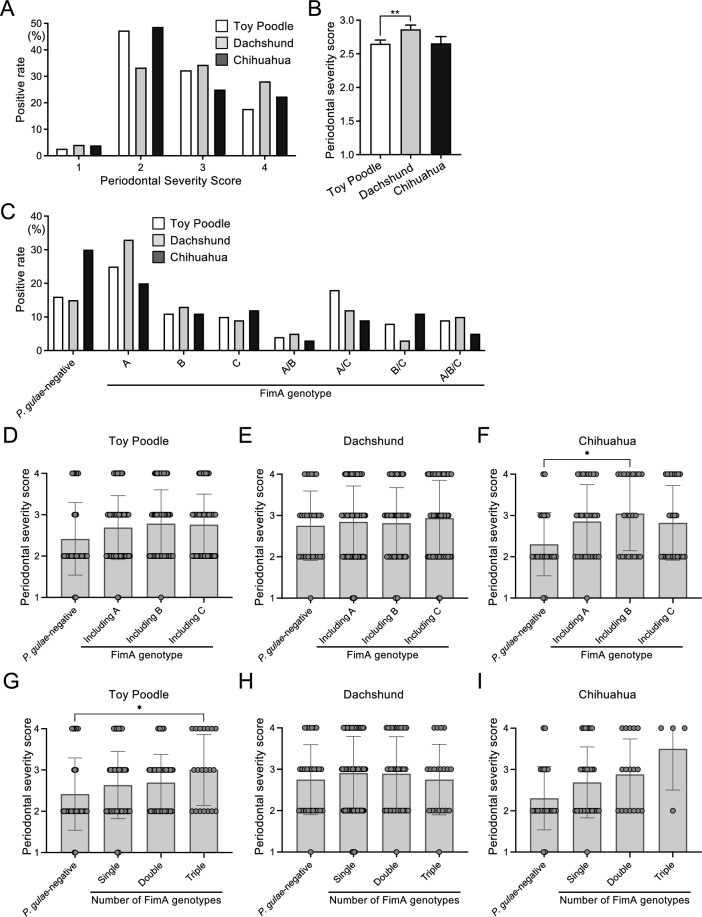
Comparison of severity of periodontal disease and FimA genotype distribution among the top three breeds: Toy Poodle, Dachshund, and Chihuahua. (**A**) Rate of dogs in each breed with each periodontal severity score. (**B**) Average periodontal severity scores of each breed. (**C**) Number of dogs in each breed with each FimA genotype distribution. Severity of periodontal disease, including FimA types A, B, and C regardless of single or multiple genotypes in Toy Poodles (**D**), Dachshunds (**E**), and Chihuahuas (**F**), and number of FimA genotypes in Toy Poodles (**G**), Dachshunds (**H**), and Chihuahuas (**I**). Each circle represents the data of one dog. Gray bars represent the mean periodontal severity score. Data expressed as the mean ± standard deviation. *P < 0.05 and **P < 0.01 between groups.

Less than 5% of dogs among the three most common breeds in the study (Toy Poodle, Dachshund, and Chihuahua) had healthy periodontal status (periodontal severity score = 1), and most had periodontal disease (Fig. 4A). The mean periodontal severity score of the three breeds was highest in the Dachshunds, and there was a significant difference between Toy Poodles and Dachshunds (*P* < 0.01) (Fig. 4B). Among Chihuahuas, dogs with *P. gulae*-negative were more common than those with any FimA genotype, and among Toy Poodles and Dachshunds, dogs with only type A were most common (Fig. 4C).

There were no significant differences in periodontal severity scores for each FimA genotype distribution in Toy Poodles, Dachshunds, and Chihuahuas (Supplementary Fig. 1). Next, *P. gulae*-positive groups were classified by having either types A, B, or C. There was no significant difference in periodontal severity scores between each group in Toy Poodle and Dachshund (Fig. 4D,E). At the same time, Chihuahua had significantly higher periodontal severity scores in type B than in *P. gulae*-negative (*P* < 0.05) (Fig. 4F). The periodontal severity score of Toy Poodles increased as the number of FimA genotypes increased, and there was a significant difference between the *P. gulae*-negative group and the triple FimA genotype group (*P* < 0.05) (Fig. 4G). There was no significant difference in periodontal severity scores between each group in Dachshund and Chihuahua (Fig. 4H,I).

### Association of missing teeth with FimA genotype distribution and periodontal severity score

Periodontal severity score 1 was concentrated in the dogs without missing teeth (Table 1). Periodontal severity score 2 applies to approximately 60% of dogs without missing teeth. The periodontal severity score 2 rate decreases in groups with more missing teeth. The periodontal severity score 2 rates in the group without missing teeth was significantly higher than the periodontal severity score 2 rates in the groups with 11–20 missing teeth and 21 or more missing teeth (*P* < 0.01). No dogs with periodontal severity scores of 1 and 2 were present in the group with 21 or more missing teeth. The periodontal severity score 4 increased as the number of missing teeth increased, and approximately 60% of dogs were in the score with 21 or more missing teeth.

**Table 1:** Periodontal severity score and *P. gulae* FimA genotype according to the number of missing teeth in small dogs.

Analysis items	Number of missing teeth			
	0 (n = 74)	1–10 (n = 69)	11–20 (n = 24)	21 (n = 21)
Age	7.6 ± 3.5	9.2 ± 3.5	10.0 ± 3.3	10.7 ± 3.3^**^
Sex (male: female)	28: 46	34: 33^a^	12: 12	7: 14
Periodontal severity score				
1	10 (13.5%)^††^	1 (1.4%)^**^	0 (0%)	0 (0%)
2	44 (59.5%)^‡‡, §§§^	34 (49.3%)^‡‡‡^	6 (25.0%)^**,^^‡‡‡^	0 (0%)^***^
3	15 (20.3%)	24 (34.8%)	10 (41.7%)	9 (42.9%)
4	5 (6.8%)^‡‡,^^§§§^	10 (14.5%)^‡‡‡^	8 (33.3%)^**,^^‡‡‡^	12 (57.1%)^***^
FimA genotype				
*P. gulae*-negative	18 (24.3%)	8 (11.6%)	2 (8.3%)	1 (4.8%)
A	18 (24.3%)	25 (36.2%)	10 (41.7%)	3 (14.3%)
B	12 (16.2%)	8 (11.6%)	2 (8.3%)	3 (14.3%)
C	4 (5.4%)*	6 (8.7%)	6 (25.0%)^*^	2 (9.5%)
A/B	4 (5.4%)	2 (2.9%)	1 (4.2%)	2 (9.5%)
A/C	8 (10.8%)	10 (14.5%)	2 (8.3%)	2 (9.5%)
B/C	6 (8.1%)	7 (10.1%)	1 (4.2%)	3 (14.3%)
A/B/C	4 (5.4%)^§^	3 (4.3%)^§^	0 (0%)^§^	5 (23.8%)^*,†,‡^

^a^Sex of 2 dogs in the number of missing teeth 1–10 group was unknown. ^*^P < 0.05, ^**^P < 0.01, and ^***^P < 0.001 versus the number of missing teeth 0; ^†^*P* < 0.05, ^††^*P* < 0.01, and ^†††^*P* < 0.001 versus the number of missing teeth 1–10; ^‡^*P* < 0.05, ^‡‡^*P* < 0.01, and ^‡‡‡^*P* < 0.001 versus the number of missing teeth 11–20; ^§^*P* < 0.05 and ^§§§^*P* < 0.001 versus the number of missing teeth more than 21.

The rates of dogs without *P. gulae* were higher in groups with fewer missing teeth, but there was no significant difference between each group. For the single FimA genotype, the rate of dogs with type C was significantly higher in the group with 11–20 missing teeth than in the group without missing teeth (*P* < 0.05). Regarding multiple FimA genotypes, the rate of dogs with all types A/B/C was significantly higher in the group with 21 or more missing teeth than in the other groups (*P* < 0.05).

### Association of mitral regurgitation with FimA genotype distribution and periodontal severity score

Dog age was significantly higher in the mitral regurgitation group than in the healthy group (*P* < 0.001) (Supplementary Table 1). Periodontal severity scores 1 and 2 were higher in the healthy group than in the mitral regurgitation group, and periodontal severity scores 3 and 4 were higher in the mitral regurgitation group than in the healthy group. However, there was no significant difference in periodontal severity scores and FimA genotype distribution between the healthy and mitral regurgitation groups.

## Discussion

In this study, 665 small breed dogs were clinically evaluated for periodontal status and FimA genotype distribution stratified by age. The results showed that periodontal disease and FimA genotype distribution increased with age. For most FimA genotype distribution patterns, dogs with that pattern had worse periodontal status than dogs negative for *P. gulae*. Furthermore, the periodontal severity, FimA genotypes distribution, and their association were different between dog breeds.

Our evaluations of periodontal tissue condition based on the method described by Araújo et al., (2019) revealed that < 5% of dogs had the lowest periodontal severity score of 1 (no significant findings), and > 95% of dogs had some periodontal disease^11^. Interestingly, all dogs under age 1 year had a score of 1, with scores of 2 (mild periodontal disease) appearing at age 1, 3 (moderate periodontal disease) appearing at age 2 years, and 4 (severe periodontal disease) appearing at age 3 years. Scores of 4 were found in dogs of all age groups above 3 years. The age-related increase in periodontal severity score mainly occurred up to age 6 years, with mean periodontal severity scores < 2.5 in all age groups from 0 to 5 years, and mean periodontal severity scores of ≥ 2.5 in all age groups above age 6 years. These results indicated that periodontal disease progresses by the age of 6 years in small breed dogs. It should be noted that there are a small number of elderly dogs (10 years or older) with a periodontal disease severity score of 1.

In recent years, researchers have focused on the timing of the establishment of bacteria that comprise the human oral biofilm^12^. Dysbiosis of the human oral microbiome is detrimental to health, leading to periodontal disease. A recent study of 225 mostly small dogs revealed that, within the first 50 months of life, the severity of periodontal disease varied according to the genotype of *P. gulae* FimA in the oral cavity^10^. This study categorized dogs into only three types of FimA genotypes: A, B, and C; however, the actual FimA genotype distribution in the oral cavity of dogs is classified into seven types (A, B, C, A/B, A/C, B/C, A/B/C). Additionally, the previous study broadly categorized dogs into three age groups (under 50 months, 50–100 months, and over 100 months). Therefore, we aimed to determine the time of settlement of each FimA genotype distribution of *P. gulae* in the oral cavity using a larger number of small dogs as subjects (n = 665). As a result, the rates for *P. gulae*-negative dogs and dogs with any FimA genotype distributions had a trend with age. This result means that the number of *P. gulae*-negative dogs decreases with age and that it is difficult to eliminate once *P. gulae* in any of the FimA genotypes is established in the oral cavity.

Without considering the number of FimA genotypes detected in the oral cavity, dogs with FimA genotypes A, B, or C of *P. gulae* have a significantly higher periodontal severity score than dogs with *P. gulae*-negative, which was consistent with a previous study^10^. In the present study, we also analyzed the number of FimA genotypes, and we found that all dogs with 1 to 3 FimA genotypes have significantly higher periodontal severity scores than the dogs negative for *P. gulae*. This result suggests that *P. gulae* colonization is related to periodontal severity score, regardless of the number of FimA genotypes. More specifically, when only one FimA genotype was detected, types B and C were significantly associated with high periodontal severity scores, which correlated with previous in vitro studies^8^. In addition, the detection of all three FimA genotypes, A, B, and C, showed the highest periodontal severity score. These results suggest that the highly pathogenic *P. gulae* affects periodontal conditions with a single FimA genotype, which is even more substantial with multiple genotypes.

The three most popular small dog breeds in the present study were Toy Poodle, Dachshund, and Chihuahua, which is consistent with a previous study^13^. Differences in periodontal disease severity and FimA genotype distribution were observed in these three dog breeds. For example, *P. gulae*-negative dogs are about twice as ordinary in Chihuahuas as in Toy Poodles and Dachshunds, and Chihuahuas may be a breed that is relatively immune to *P. gulae* infection. Furthermore, the FimA genotype distribution was not related to the periodontal severity score, although Dachshund had the highest periodontal disease severity score among the three dog breeds. From this, the periodontal condition of Dachshunds may be significantly influenced by factors other than *P. gulae* infection. Our study revealed that periodontal disease severity and FimA genotype distribution differ between dog breeds, and the differences in periodontal disease severity and FimA genotype distribution by breed should be clarified in more detail.

This study included a random sample of small breed dogs that visited Japanese veterinary clinics in a specific region. However, it has recently become clear that human subjects with special systemic conditions due to disease or congenital abnormalities develop a unique oral flora^14,15^. Additionally, oral status varies widely among countries with different economic conditions^16,17^. Therefore, future studies on small breed dogs may need to focus on those with systemic diseases and congenital abnormalities in more detail, as well those bred in different countries and regions.

Our previous human clinical study evaluated the presence and the amount of dental plaque^18^. In that study, we assessed the presence and the amount of plaque using a dental plaque-disclosing agent. Still, dental plaque-disclosing agents are generally not used in clinical practice targeting small dogs. Additionally, since it is impossible to remove plaque in small dogs through daily oral cleaning in the same manner as in humans, we did not evaluate the presence and the amount of dental plaque.

In summary, our results suggest that periodontal disease in small breed dogs progresses gradually from birth, and that chronic periodontal disease develops within several years. Additionally, *P. gulae* bacteria with various FimA genotypes became established in the oral cavity over a period of several years after birth. Dogs positive for *P. gulae* had more severe periodontal disease than dogs negative for *P. gulae*, especially in dogs with specific FimA genotypes such as types B, C, A/B/C. Furthermore, major dog breeds differed in periodontal disease severity and FimA genotype distribution. To prevent periodontal disease in small breed dogs, we propose that it is important to prevent the establishment of oral infection with highly pathogenic FimA *P. gulae* bacteria at an early age, and that identification of the FimA genotype is effective for determining the risk of periodontal disease.

## Methods

### Subjects and oral sample collection

**Figure 5:**
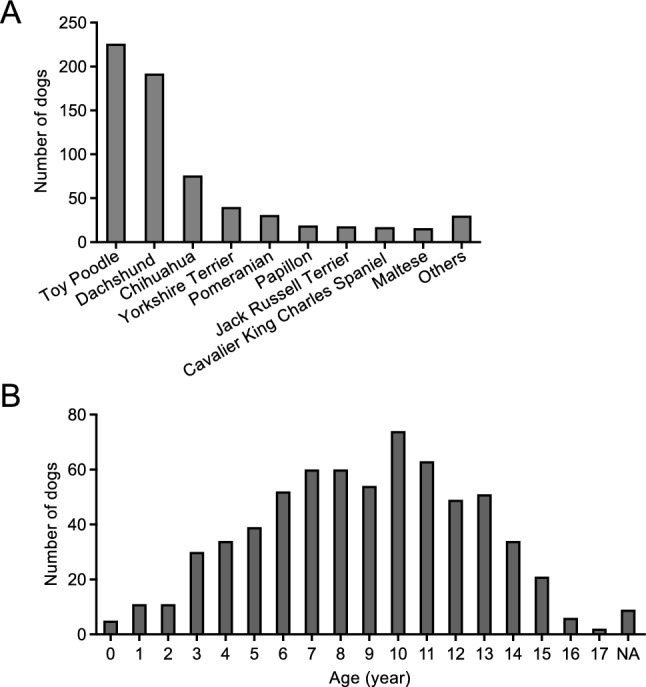
Breed and age distribution of the 665 small breed dogs. Breeds (**A**) and age distribution (**B**) of dogs included in the study. NA indicates dogs of unknown age.

A total of 665 small breed dogs (331 males, 231 sterilized; 325 females, 234 sterilized) with a median age of 9 years (range: 0–17 years) who were seen at 77 veterinary clinics in Japan between March 2015 and May 2022 were included. The number and age distributions of each breed of dog are shown in Fig. 5 and Supplementary Fig. 2. Breeds with fewer than 10 dogs (Italian Greyhound, Bichon Frise, Toy Manchester Terrier, Norfolk Terrier, Miniature Pinscher, Cairn Terrier, Sealyham Terrier, Norwich Terrier, Bedlington Terrier, Wire Fox Terrier) were classified as other (n = 30). Systemic medical history included none (n = 456), mitral regurgitation (n = 65), renal failure (n = 17, of which four dogs had concurrent mitral regurgitation), and hypothyroidism (n = 13, of which two dogs concurrent mitral regurgitation, and one dog concurrent renal failure), other diseases (n = 99, all diseases were less than n = 10), and unknown (n = 22). Oral swab specimens were collected from the gingival margin of the maxillary right or left canine and fourth premolar using a micro brush (Microapplicator fine, FEED Corporation, Yokohama, Japan), as previously reported^19,20^. In counting missing teeth, we only counted permanent ones and did not include deciduous ones. All study protocols were conducted in full adherence to the principles of the Declaration of Helsinki and were approved by the Animal Care and Use Program of Azabu University (Approval No. 200318–1). All owners were informed of the content of the study and gave written informed consent for their dogs to participate.

### Evaluation of periodontal conditions

Periodontal severity scores were determined by evaluation of the gingival margin of the maxillary right or left canine and fourth maxillary premolar, using a previously described method^20,21^. For each dog, periodontal severity scores were evaluated visually as follows: (1) no significant findings; (2) mild periodontal disease—gingival swelling, gingival regression, and halitosis; (3) moderate periodontal disease—exposure of root, spontaneous bleeding, and tooth loss; and (4) severe periodontal disease—furcation involvement and fistula formation.

### Detection of *P. gulae* and FimA genotypes in clinical samples

Distributions of *P. gulae* and FimA genotypes were determined using previously developed polymerase chain reaction (PCR)-based methods^5,8^. Bacterial DNA was extracted from each oral specimen using a Gentra Puregene Yeast/Bact. Kit B (Qiagen, Hilden, Germany). First, each bacterial DNA specimen was used as template for PCR using a universal primer set targeting 16S rRNA genes^22^ to confirm successful DNA extraction (Table 2). Second, each specimen was amplified using respective specific primer sets to determine the *P. gulae* and FimA genotypes^8,9,23^. Amplification reactions were performed with 1 µL template solution and Ex *Taq* DNA Polymerase (Takara Bio. Inc., Otsu, Japan) in a total volume of 20 µL with the following cycling parameters: initial denaturation at 95 °C for 4 min; 30 cycles of 95 °C for 30 s, 60 °C for 30 s, and 72 °C for 30 s; and a final extension at 72 °C for 7 min. PCR products were separated by electrophoresis on a 1.5% agarose gel in Tris–acetate-EDTA buffer. The gels were stained with 0.5 μg/mL ethidium bromide and photographed under ultraviolet illumination.

**Table 2:** PCR primers used in the present study.

Specific primer set	Sequence (5′–3′)	References
Universal primer		
PA	AGA GTT TGA TCC TGG CTC AG	^22^
PD	GTA TTA CCG CGG CTG CTG	
Detection of *P. gulae*	TTG CTT GGT TGC ATG ATC GG	^19^
	GCT TAT TCT TAC GGT ACA TTC ACA	
Specification of *fimA* type		
Type A *fimA*		
Pgfim-AF	TGA GAA TAT CAA ATG TGG TGC AGG CTC ACG	^8^
Pgfim-AR	CTT GCC TGC CTT CAA AAC GAT TGC TTT TGG TAA GAT	
Type B *fimA*		
Pgfim-BF	TGA AGT GAA GAT GAG GGA TTC TTA TGT	^23^
Pgfim-BR	ATT TCC TCA GAA CTC AAA GGA GTA CCA TCA	
Type C *fimA*		
Pgfim-CF	CGA TTA TGA CCT TGT CGG TAA GAG CTT GGA	^8^
Pgfim-CR	TGT GGC TTC GTT GTC GCA GAA TCC GGC ATG	

### *Porphyromonas gulae* strains

*Porphyromonas gulae* strains ATCC 51,700 (FimA type A), D040 (FimA type B), and D049 (FimA type C) were selected from our laboratory stock culture collection^7,8,23^. Bacterial cells were grown anaerobically at 37 °C for 24 h in trypticase soy broth supplemented with yeast extract (1 mg/mL), hemin (5 μg/mL), and menadione (1 μg/mL), as previously described^24^. Genomic DNA was extracted from the bacterial culture medium and used as a positive control for PCR analysis.

### Statistical analysis

Statistical analyses were conducted using GraphPad Prism 9 (GraphPad Software Inc., La Jolla, CA, USA). For comparisons of periodontal severity scores in small dogs of each age and breed and comparison of periodontal severity scores between different FimA genotypes, we used the Kruskal–Wallis test for nonparametric analysis, followed by the Dunn test for multiple comparisons. Age adjustments were made between groups of different ages. The test for linear trend was performed using one-way ANOVA. Chi-square tests with Bonferroni’s correction were used for comparisons in each number of missing teeth group. Comparisons between healthy and mitral regurgitation groups were performed using Fisher’s extract test. Differences were considered statistically significant at *P* < 0.05.

### Supplementary Information

Supplementary Information.

## Data Availability

The datasets used and/or analysed during the current study available from the corresponding author on reasonable request.
