# Supplementary material for: Investigation of periodontal disease development and Porphyromonas gulae FimA genotype distribution in small dogs
Source: Sci Rep. 2024 Mar 4;14:5360. doi: 10.1038/s41598-024-55842-8 (PMC10912432; doi:10.1038/s41598-024-55842-8)
Supplement: Supplementary file 1 — Supplementary Information. [file 41598_2024_55842_MOESM1_ESM.pdf]

Investigation of periodontal disease development and *Porphyromonas gulae* FimA genotype distribution in small dogs

Junya Yasuda, Hidemi Yasuda, Ryota Nomura, Saaya Matayoshi, Hiroaki Inaba, Enrique Gongora, Naoki Iwashita, So Shirahata, Noriyuki Kaji, Tatsuya Akitomo, Chieko Mitsuhata, Jumpei Uchiyama, Tomoki Fukuyama, Michiyo Matsumoto-Nakano, Kazuhiko Nakano & Masaru Murakami

Supplementary Table 1. Periodontal severity score and *P. gulae* FimA genotype in healthy and mitral regurgitation dogs.

	Analysis items	Healthy (n=425)	Mitral regurgitation (n=65)	*P* value
	Age	7.9 ± 3.4[a]	11.5 ± 2.4[a]	<0.001
	Sex (male: female)	208: 210	37: 27	0.283
Periodontal severity score	1	21 (4.9%)	1 (1.5%)	0.337
	2	184 (43.3%)	20 (30.8%)	0.060
	3	132 (31.1%)	26 (40.0%)	0.156
	4	88 (20.7%)	18 (27.7%)	0.260
FimA genotype	*P. gulae*-negative	73 (17.2%)	15 (23.1%)	0.297
	A	111 (26.1%)	14 (21.5%)	0.541
	B	47 (11.1%)	10 (15.4%)	0.302
	C	50 (11.8%)	5 (7.7%)	0.404
	A/ B	14 (3.3%)	0 (0.0%)	0.233
	A/ C	59 (13.9%)	10 (15.4%)	0.705
	B/ C	33 (7.8%)	5 (7.7%)	>0.999
	A/ B/ C	38 (8.9%)	6 (9.2%)	>0.999

[a]The ages of 7 dogs in the healthy group and 1 dog with mitral regurgitation were unknown.

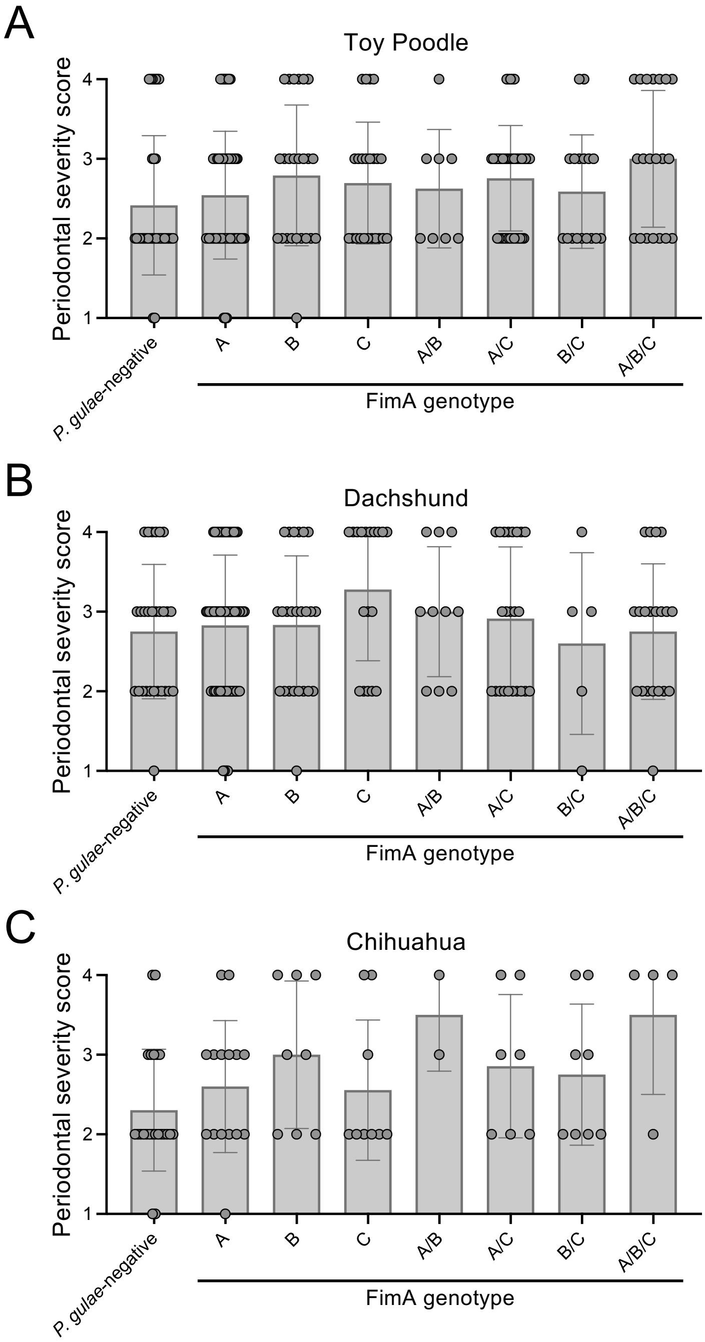

Supplementary Fig. 1 Periodontal severity scores in FimA genotype distribution among the three dog breeds. Toy Poodles (A), Dachshunds (B), and Chihuahuas (C). Each circle represents the data of one dog. Gray bars represent the mean periodontal severity score. Data expressed as the mean ± standard deviation.

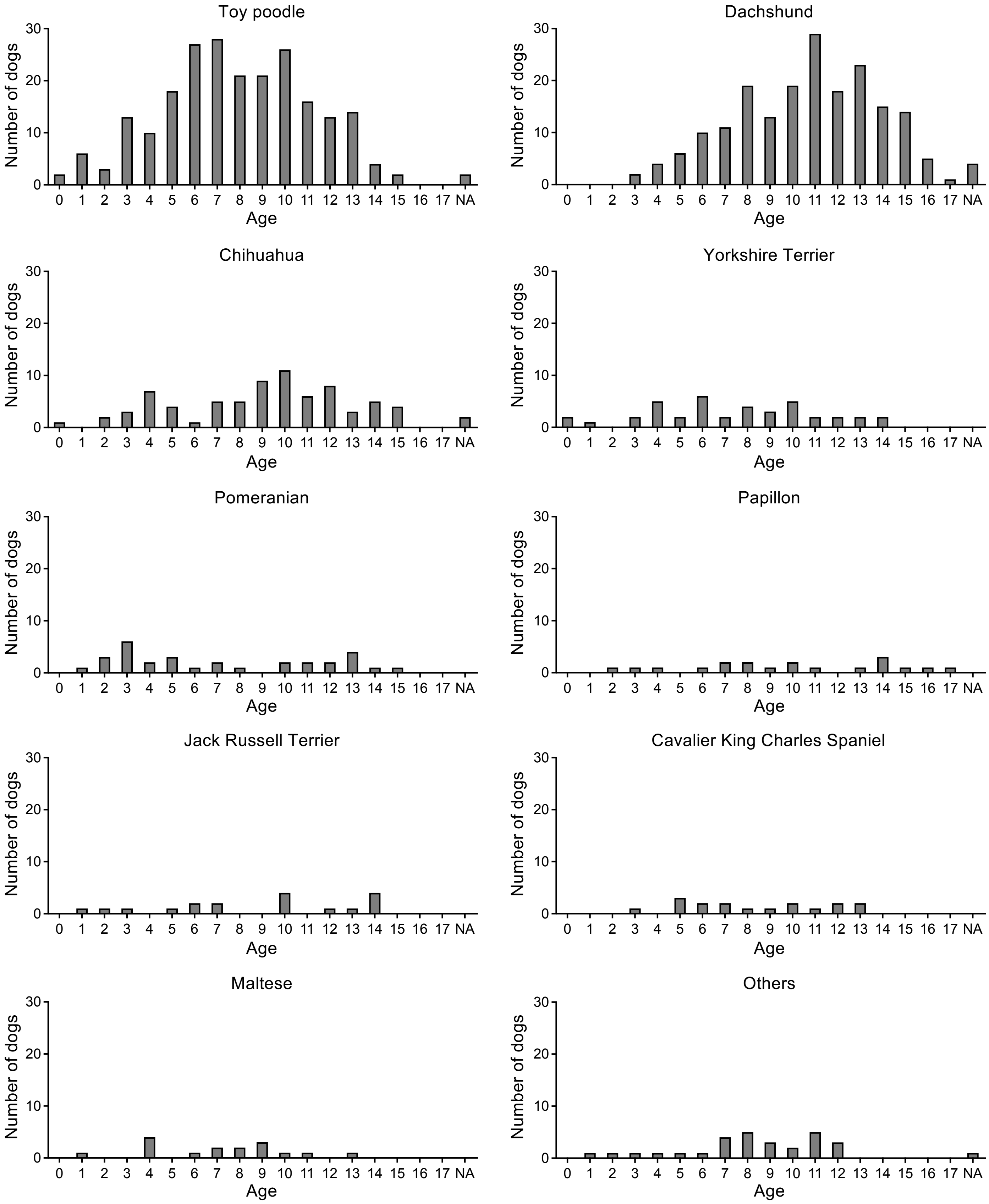

Supplementary Fig. 2 Age distribution of each small dog breed.
